# Effect of Pre-Conditioning Temperature and Method of Curing on the Shear Bond Strength of Dual-Cure Composite Cements to Dentin

**DOI:** 10.3390/ma19040718

**Published:** 2026-02-13

**Authors:** Joanna Giełzak, Agata Szczesio-Włodarczyk, Kinga Bociong

**Affiliations:** 1 Department of Prosthodontics, Medical University of Lodz, 92-213 Łódź, Poland; joanna.gielzak@umed.lodz.pl; 2 Laboratory of Materials Research, Medical University of Lodz, 92-213 Łódź, Poland; 3 Department of General Dentistry, Medical University of Lodz, 92-213 Łódź, Poland

**Keywords:** prosthetic dentistry, storage temperature, resin cements, dual-cured composite cements, chemically cured, shear bond strength

## Abstract

Dual-cure composite cements are an important element of modern dental prosthetics, enabling a stable and long-lasting bond between prosthetic restorations and tooth tissues. Thanks to the combined mechanism of chemical- and light-curing polymerization, they are characterized by high clinical versatility. Despite their wide application, the impact of storage/pre-conditioning temperature on the mechanical properties of dual-cure composite cements remains unclear. The study evaluated the shear bond strength (SBS) of the bond between four dual cements—Bifix Hybrid Abutment (VOCO GmbH, Cuxhaven, Germany), MaxCem Elite (Kerr Corporation, Orange, CA, USA), EnaCem HF (Micerium, Avegno, Italy), and Multilink Automix (Ivoclar Vivadent, Schaan, Lichtenstein)—and dentin depending on their storage temperature (25 °C or 50 °C) and curing method. The tests were carried out on extracted human permanent teeth. The cements were divided into two temperature groups—stored for 7 days at 25 °C or stored for 7 days at 50 °C—and then each of these two temperature groups was divided into two groups—light- and chemically cured (dual-cured, LC) and chemically cured only (CC). Dual-cured cements showed higher shear bond strength at 25 °C. Storage at 50 °C lowered the SBS values, especially for the purely chemically bound cements. LC Bifix Hybrid Abutment achieved the highest SBS at 25 °C, but at 50 °C its properties deteriorated. EnaCem HF showed higher strength at a lower temperature; MaxCem Elite was stable at both temperatures, whereas Multilink Automix showed lower SBS at 50 °C. The study showed that the chemical composition of cements, especially the presence of a benzoyl peroxide (BPO) initiating system, can play a key role in their SBS when bonded to teeth tissue and stability at different storage temperatures. MaxCem Elite showed the best resistance to temperature changes—it achieved the highest temperature stability in both temperature groups.

## 1. Introduction

Dual-cure composite cements are an important component of modern dental prosthetics, enabling an effective, permanent bond between the prosthetic restoration and tooth tissues. However, various factors may influence their performance, including the curing process [1,2]. In prosthetic restorations, there is a high risk of insufficient light activation due to the restorative material’s type and thickness, as well as limited access of the curing light to internal restoration surfaces. Studies have shown that increasing the thickness and opacity of ceramics leads to incomplete polymerization and lower cross-linking density, reducing the cement’s strength and bond durability [3,4,5]. The popularity of dual-cured resin cements results from the combination of two polymerization mechanisms—chemical and light curing—which means that they can be versatile—used both in places with good light access and where light penetration is limited [4]. Light-curing polymerization depends on the presence of photoinitiators, e.g., camphorquinone, while chemical curing occurs thanks to peroxide initiators and amines. The effectiveness of both these mechanisms is crucial for the complete conversion of dual-cured composite cements and ultimately determines the clinical performance [1]. Arrais et al. [6] demonstrated that the method of activating dual-cure cements has a significant impact on their chemical and mechanical properties. Another study indicated the superiority of dual-cure mechanism over photo-activation or self-curing alone [7]. It was also pointed out that the degree of conversion of the monomer system increases with light curing [8]. In turn, a recent systematic review by D’Alessandro et al. [9] compared conventional light curing with the “tack–cure” technique, which involves brief light exposure to facilitate removal of excess cement before complete polymerization. This method improves clinical handling and provides materials with mechanical performance comparable to, or even better than, that of the conventional polymerization method. But it should be an underlying necessity of connecting this polymerization mode with the type of resin cement.

Another factor that may influence the mechanical and adhesive properties of dual-cure cements is application and storage temperature. Current research predominantly explores the short-term thermal effects on resin-based materials. Evidence from multiple studies indicates that preheating composite resins reduces their viscosity, promotes higher conversion and microhardness, and improves marginal fit in restorative procedures [10]. Maucoski et al. [11] measured the in vitro effect of different temperatures (23, 32, or 37 °C) and the use of a primer on the setting time and degree of conversion of a novel dual-cure self-adhesive cement. They indicated that temperature increases the degree of conversion and that light exposure has an impact on polymerization rate.

Storing resin materials at inappropriate temperatures can alter their physical and chemical stability [12]. It has been shown that water-based dental luting agents subjected to increased temperature and humidity exhibit variations in their properties over 84-month storage [13]. Sadr et al. [14] focused on self-etching adhesives and demonstrated that prolonged storage at elevated temperatures (60 weeks at 37 °C) reduced bond strength and pH values due to hydrolytic degradation. Such storage instability issues are not unique to acidic monomers—hydrophilic components such as HEMA also show susceptibility to temperature-induced degradation [14]. These results suggest that similar mechanisms may occur in dual-cure cements, particularly in self-adhesive or new universal resin cements containing functional monomers, which may affect their adhesive properties. Another study compared the effects of mixing and storage errors in two resin-based dental cements. It was found that the failure risk of glass–ceramic crowns remained stable across a wide range of mixing proportions. However, improper storage conditions adversely affected the chemical polymerization capacity of these cements [15].

Based on the above, it should be emphasized that in the literature there is still a lack of precise data on the effect of shelf storage temperature on the strength and durability of the cement–tooth bond and its mechanical stability. A previous study by Giełzak et al. [16] examined the influence of storage temperature on selected mechanical parameters of dual-cure composite cements. However, the effect of this factor on the adhesive bond to dentin remains unclear. Therefore, it is crucial to determine how different storage temperatures affect the shear bond strength of resin cements; in particular, the chemical-curing capacity should be evaluated.

This research, therefore, provides novel data on how storage-related thermal factors can affect the bonding performance of dual-cure resin cements. In this study, temperatures of 25 °C and 50 °C were used to represent two clinically relevant storage scenarios. The value of ~25 °C corresponds approximately to typical room temperature and is consistent with manufacturers’ recommendations for the storage of resin materials. On the other hand, a temperature of 50 °C was selected to simulate potential overheating during transport or storage in non-air-conditioned environments and is also frequently used in studies on preheating of dental materials, as it may enhance material density and promote a higher degree of conversion [17,18,19,20]. This study investigated the effect of elevated pre-conditioning temperature on the bonding efficiency of resin cements polymerized via chemical (self-cure) and dual-cure modes. The first null hypothesis was that the pre-conditioning temperature of dual-cure cements influences their shear bond strength (SBS) to dentine. The second one was that there are no differences in SBS between light-cure and chemical-cure cements bonded to dentine.

## 2. Materials and Methods

Four types of dual-cure composite cements were used in the study: Bifix Hybrid Abutment (VOCO, Cuxhaven, Germany), MaxCem Elite (Kerr Corporation, Orange, CA, USA), EnaCem HF (Micerium, Avegno, Italy), and Multilink Automix (Ivoclare Vivadent AG, Schaan, Lichtenstein). The chemical compositions of the investigated cements are presented in Table 1. These cements were stored at two different temperatures: 25 °C (room temperature) and 50 °C for a period of 7 days before the tests were carried out. Each cement was divided into two groups depending on the curing method: cements bonded chemically and by light polymerization (LC, dual-cured) and cements bonded only chemically, without light activation (CC). The selection and storage of teeth were carried out in accordance with the ISO 11405:2015 [21] guidelines for testing the adhesion of dental materials to dental hard tissue [18]. Human teeth were used as a substrate, without active caries and without visible fillings. These teeth were then prepared for testing by removing the enamel and polishing the dentin surface. The teeth used in the study were obtained from patients of the Maxillofacial Surgery Clinic at the university hospital and were removed for clinical reasons, with surgical or orthodontic indications unrelated to this study. The final number of samples included in the statistical analysis was n = 9 for all test groups. Only samples that were properly prepared and not damaged prior to the strength test were included in the statistical analysis. The study was conducted in accordance with the opinion of the Bioethics Committee of the Medical University of Lodz regarding this research project (opinion no. RNN/288/23/KE, dated 12 December 2023).

The dentin surfaces were etched with 37% orthophosphoric acid before cementation for 15 s. After rinsing with water and drying, a bonding system—a single-component, light-curing etch-and-rinse adhesive (Solo Bond Plus, VOCO GmbH, Cuxhaven, Germany)—was applied according to the manufacturer’s instructions. The application procedures for the materials used in this study, as well as their types and manufacturers, are presented in Table 2. The composite cement was applied into a silicone mold (diameter of 3 mm and height of 3 mm) directly on the prepared dentine surface using a self-mixing syringe tip. The material was applied in a controlled and repeatable manner by a single operator. After applying the cements to the dentin surface and carrying out the polymerization process (if applicable), all samples, regardless of the curing method, were stored in deionized water for 24 h at a temperature of 37 °C before testing. In chemically cured (CC) groups, samples were left to self-polymerize without light activation under controlled laboratory conditions (room temperature 23 °C, without light access), in accordance with the manufacturers’ recommendations. All samples remained undisturbed for the time necessary to complete the chemical-curing process (standardized for all materials—10 min) before further storage and strength testing. In order to standardize the procedure, in the LC group, the material was left for 90 s before light activation to initiate the chemical process.

The shear bond strength of the cement to dentin was determined using a Zwick/Roell Z010 testing machine (Zwick-Roell GmbH & Co.KG, Ulm, Germany). The load was applied at 1 mm/min, and the maximum force was recorded when the cement layer detached from the dentin. The results were recorded in megapascals (MPa). The data obtained in the study were subjected to statistical analysis. The normality of the distribution was assessed using the Shapiro–Wilk test. After assessing normality with the Shapiro–Wilk test, the data were analyzed using either parametric tests (one-way ANOVA with an appropriate post hoc procedure) or non-parametric tests (Kruskal–Wallis test with multiple comparisons of mean ranks), depending on the distribution. The level of statistical significance was set at *p* < 0.05.

## 3. Results

**Figure 1:**
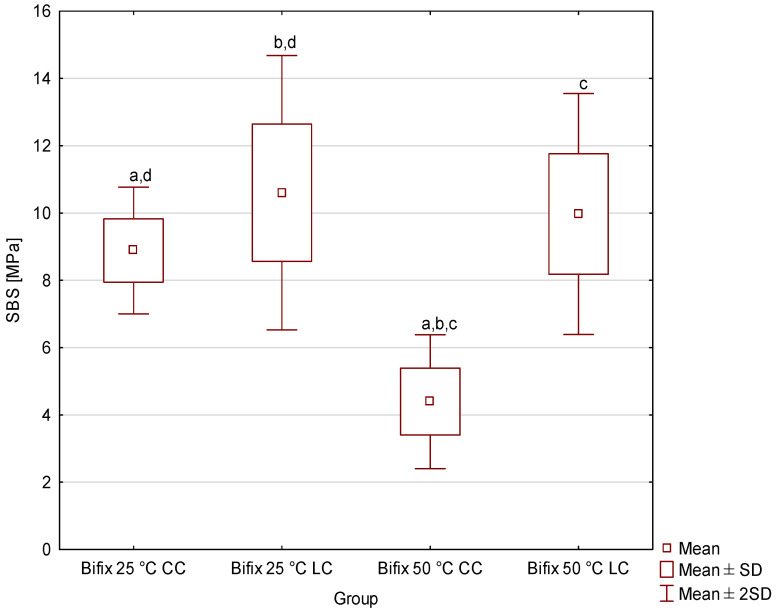
Mean and standard deviation of shear bond strength (SBS) values of Bifix Hybrid Abutment cement as a function of storage temperature and polymerization mode (LC—dual-cure polymerization; CC—chemi-calcure polymerization). Groups marked with the same lowercase letters (a–d) indicate statistically significant differences between them (*p* < 0.05); groups marked with different letters are not significantly different.

**Figure 2:**
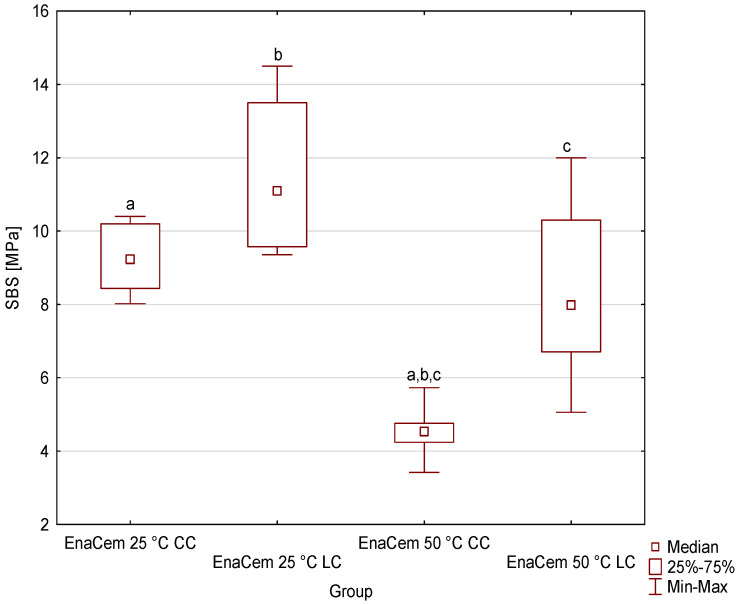
Medians with quartile deviations of shear bond strength (SBS) values of EnaCem HF cement as a function of storage temperature and polymerization mode (LC—dual-cure polymerization; CC—chemical-cure polymerization). Groups marked with the same lowercase letters (a–c) indicate statistically significant differences between them (*p* < 0.05); groups marked with different letters are not significantly different.

**Figure 3:**
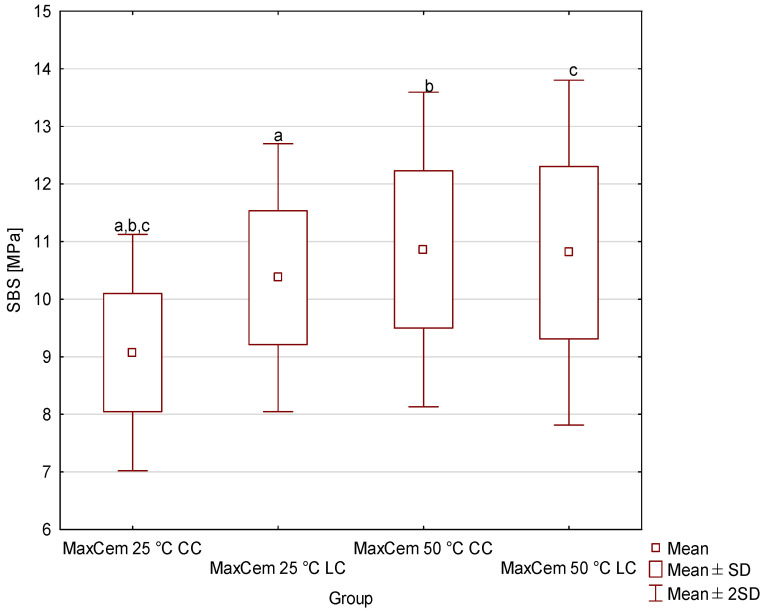
Mean and standard deviation of shear bond strength (SBS) values of MaxCem Elite cement as a function of storage temperature and polymerization mode (LC—dual-cure polymerization; CC—chemical-cure polymerization). Groups marked with the same lowercase letters (a–c) indicate statistically significant differences between them (*p* < 0.05); groups marked with different letters are not significantly different.

**Figure 4:**
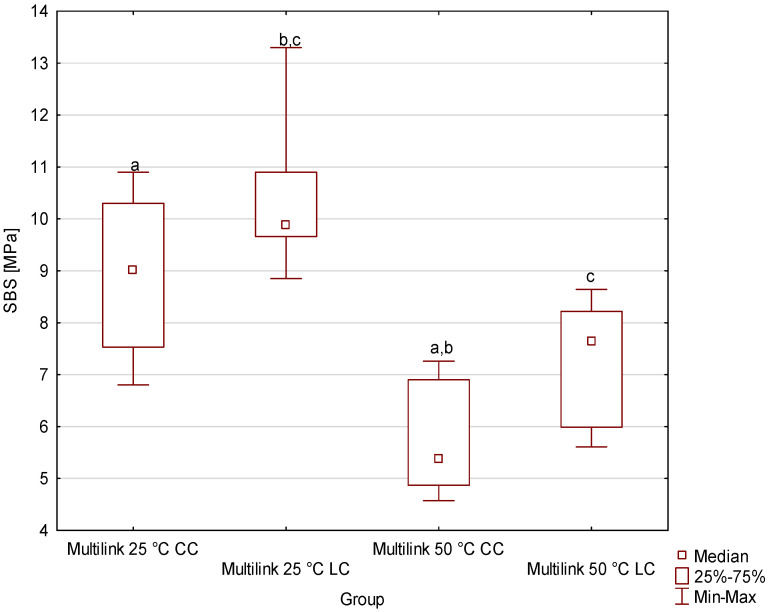
Medians with quartile deviations of shear bond strength (SBS) values of Multilink Automix cement as a function of storage temperature and polymerization mode (LC—dual-cure polymerization; CC—chemical-cure polymerization). Groups marked with the same lowercase letters (a–c) indicate statistically significant differences between them (*p* < 0.05); groups marked with different letters are not significantly different.

Figure 1, Figure 2, Figure 3 and Figure 4 shows the shear bond strength (SBS) values of the four tested dual-cure composite cements, taking into account the effects of storage temperature and the polymerization mode used. Descriptive statistics (minimum, maximum, mean, standard deviation and median with interquartile range of SBS) are given in the Supplementary Materials (Table S1). For Bifix Hybrid Abutment (Figure 1), SBS values ranged from 4.39 MPa (50 °C, CC) to 10.61 MPa (25 °C, LC); for EnaCem HF (Figure 2), from 3.42 MPa (50 °C, CC) to 14.5 MPa (25 °C, LC); for MaxCem Elite (Figure 3), from 9.07 MPa (25 °C, CC) to 10.86 MPa (50 °C, CC); and for Multilink Automix (Figure 4), from 5.38 MPa (50 °C, CC) to 9.88 MPa (25 °C, LC). In each material, the highest SBS values were noted for the subgroups cured with light assistance (LC) at 25 °C, except for MaxCem Elite, which reached its highest value in the 50 °C CC subgroup, while the lowest values were observed in the 50 °C CC subgroups for Bifix Hybrid Automix, EnaCem HF, and Multilink Automix. The test results showed statistically significant differences in SBS between the dual-cure cements analyzed, depending on the storage temperature used. Particularly pronounced differences were observed for cements stored at 50 °C, where in three out of four cases (Bifix Hybrid Abutment, EnaCem HF, and Multilink Automix) the purely chemical-cured (CC) specimens showed significantly lower shear bond strengths compared to those stored at 25 °C. The failure mode was visually evaluated (Table S2); in almost all samples, it was sheared adhesively.

For Bifix Hybrid Abutment cement, it was shown that the highest SBS was achieved in the Bifix Hybrid Abutment 25 °C LC group, while the lowest values were recorded for Bifix Hybrid Abutment 50 °C CC (*p* < 0.000001). Statistically significant differences were recorded between Bifix Hybrid Abutment 50 °C CC and Bifix Hybrid Abutment 25 °C CC (*p* = 0.000000); Bifix Hybrid Abutment 50 °C CC and Bifix Hybrid Abutment 25 °C LC (*p* = 0.000000); Bifix Hybrid Abutment 50 °C CC and Bifix Hybrid Abutment 50 °C LC (*p* = 0.000000); and Bifix Hybrid Abutment 25 °C CC and Bifix Hybrid Abutment 25 °C LC (*p* = 0.022560).

In the case of EnaCem HF cement, statistical analysis showed a lack of homogeneity of variance, which necessitated the use of non-parametric tests. The results indicate that the lowest SBS value was achieved for EnaCem HF 50 °C CC, and significant differences were found between EnaCem HF 50 °C CC and EnaCem HF 25 °C CC (*p* = 0.007954); EnaCem HF 50 °C CC and EnaCem HF 25 °C LC (*p* = 0.000018); and EnaCem HF 50 °C CC and EnaCem HF 50 °C LC (*p* = 0.029941).

A parametric analysis was carried out for MaxCem Elite cement, which showed significant differences between the binding variants and the storage temperature. MaxCem Elite 50 °C LC cement showed higher strength than the other variants. Statistically significant differences were found between MaxCem Elite 25 °C CC and MaxCem Elite 25 °C LC (*p* = 0.038070); MaxCem Elite 25 °C CC and MaxCem Elite 50 °C CC (*p* = 0.005489); and MaxCem Elite 25 °C CC and MaxCem Elite 50 °C LC (*p* = 0.006965).

In the case of Multilink Automix cement, statistical analysis indicated a lack of normality, necessitating the use of non-parametric tests. The lowest SBS value was recorded for Multilink Automix 50 °C CC. Statistically, significant differences were shown between Multilink Automix 50 °C CC and Multilink Automix 25 °C CC (*p* = 0.005804); Multilink Automix 50 °C CC and Multilink Automix 25 °C LC (*p* = 0.000021); and Multilink Automix 50 °C LC and Multilink Automix 25 °C LC (*p* = 0.018204).

## 4. Discussion

The choice of the topic concerning the impact of storage temperature on the properties of composite cements with a dual setting mechanism was due to the growing importance of these materials in everyday clinical practice and the limited number of available analyses taking this factor into account. Storage temperature is a variable that can realistically affect the clinical performance of resin cements, yet it remains underexplored in the literature. The values of 25 °C and 50 °C were adopted as representative of two extreme scenarios: 25 °C was selected as it corresponds to typical room temperature, which falls within the storage conditions commonly recommended for resin-based dental material, and elevated temperatures that may occur during transport or long-term storage of materials in non-air-conditioned rooms, especially during the summer. Low-temperature conditions were not considered in this study, as its aim was to focus on room temperature and elevated storage temperatures, which are typical and potentially adverse for the transport and storage of resin cements. In addition, 50 °C was also frequently used as an elevated temperature in research, which primarily involved preheating resin materials to improve their properties [10,17,18,19,20]. A limitation of the present study is the absence of data regarding dependence between low-temperature cement storage and its SBS with dentin; future work should investigate this aspect.

In addition, it was decided to divide the cements into two subgroups: light- and chemically activated (LC, dual-cure) and chemically cured only (CC, self-cure). The results of this study indicate that storage temperature can influence the shear bond strength (SBS) of dual-cure composite cements. A statistically significant reduction in SBS at 50 °C was observed for three materials in the chemically cured (CC) group, whereas for MaxCem Elite, the lowest SBS values were found at 25 °C. These findings suggest that the effects of temperature on bonding cannot be generalized across all materials or curing mechanisms. Consequently, the stated hypothesis could not be fully rejected.

Most of the research available in the literature has focused on the short-term impact of elevated temperature on the properties of resin-based materials. A systematic review by Pires Lopes et al. [22] indicates that preheating resin-based materials prior to application enhances their flow characteristics and promotes faster polymerization. Nevertheless, in the case of resin cements, the available evidence is inconclusive, and the potential advantages of thermal conditioning continue to be a subject of ongoing discussion [22]. França et al. [23] demonstrated that preheating dual-cure resin cements to 50 °C resulted in a higher degree of conversion, although ultimate tensile strength varied with the curing mode. Other authors have observed that heating influenced only water sorption and solubility, without affecting hardness, tensile strength, or the degree of conversion. The authors claimed that rapid cooling of the material during hand-mixing and manipulation could explain the obtained results [24]. The effects of pre-cure temperature on the bonding potential of resin cement have also been investigated. Heating Panavia F 2.0 to 55–60 °C prior to use resulted in a lower push-out strength of fiber-reinforced root canal fillings than that obtained with the material used at room temperature [25]. Another study assessed the effect of heating dual-cure cements on the microtensile bond strength (mTBS) between indirect restorations and dentine. The analysis showed that, for the Variolink II system, heating the material to 50 °C resulted in a significant increase in mTBS values regardless of the polymerization mode used. In contrast, in the Calibra cement groups, no statistically significant differences were observed between values obtained at 25 °C and 50 °C. It has been suggested that elevated temperature may alter the polymerization process of resin cements depending on their benzoyl peroxide (BPO) content. As BPO decomposes more rapidly at higher temperatures, more free radicals are generated, thereby accelerating curing and increasing the degree of conversion. Yet when the BPO concentration is high, as in Calibra, the setting reaction may proceed too quickly, leading to premature cross-linking and the formation of internal stress at the adhesive interface [20,26]. Cantoro et al. [27,28] investigated different dental cements that had been stored for 24 h at 4, 24, 37, and 60 °C. Their results showed that maintaining the materials at 60 °C did not enhance bond strength to dentin and, in several cases, premature polymerization rendered the materials unusable. These observations suggest that exposure to elevated temperature can lead to irreversible changes within the material’s structure, ultimately impairing its clinical performance.

However, it is important to note the differences in the research protocol used in pre-heating studies. In most of the cited research, the heating occurred briefly before the usage of the cements. In our study, the experimental conditions differed: the cements were stored at 50 °C for 7 days before use. This procedure was intended to simulate the potentially adverse storage and transport conditions that materials may actually encounter in clinical practice. While short-term heating immediately before application may in some cases improve the material’s performance, prolonged exposure to elevated temperatures may lead to premature activation of polymerization initiators, and, consequently, a reduction in SBS values, as demonstrated in this study for chemically cured samples. Studies involving prolonged storage of resin cements at elevated temperatures have confirmed our observations. Klaisiri et al. [29] examined the influence of storing composite cements at 4, 25, and 40 °C for three months. Their findings indicated that storage at 40 °C resulted in a marked decrease in bond strength to dentin. Similarly, Ozer et al. [30] reported that the bond strength of self-adhesive resin cements to dentin was higher when the materials were stored for an extended period (three months) under refrigeration (6 °C) compared with room temperature (19 °C).

The observations from this study, supported by previous reports [27,28,29,30], indicate that composite cements are sensitive to temperature variations. This factor may pose a risk in clinical practice and should be carefully considered during the transport and storage of materials, particularly as ambient temperatures rise with global warming. When comparing the present results with those in the literature, it is evident that the temperature effect largely depends on the resin cement’s chemical composition. The most critical and thermally unstable component appears to be benzoyl peroxide (BPO), which plays a key role in the chemical-curing mechanism. Studies have shown that BPO stability decreases with increasing temperature—particularly above 45 °C—regardless of whether it is incorporated into paste or powder. Under such conditions, spontaneous decomposition of BPO or premature polymerization within the paste may occur, leading to a reduction in bond strength [20,31]. The effect of elevated temperature on the dual-cure polymerization mechanism was not as pronounced as in the chemically cured specimens, which supports the assumption that benzoyl peroxide is the main factor responsible for the deterioration of the cement’s properties. Nevertheless, it should be emphasized that in clinical conditions, light penetration is often limited. Under such circumstances, the chemical component of the polymerization process becomes crucial, as it ensures adequate curing where light activation is insufficient [6,32]. Any reduction in the efficiency or stability of this chemically initiated reaction may therefore directly affect the final durability and integrity of the prosthetic restoration.

In contrast, the material containing a different chemical initiator system (MaxCem Elite) [33] did not exhibit a decline in SBS values when stored at elevated temperatures. In fact, a slight improvement in bond strength to dentin was observed (Figure 3). This finding suggests that the choice of cement formulation becomes particularly important in regions with high ambient temperatures, where transportation and storage conditions may cause irreversible changes within the material syringe.

Another factor to consider is the thermal stability of acid-functionalized monomers, which are incorporated into modern self-adhesive and so-called “truly universal” resin cements [34,35]. These monomers may undergo hydrolysis or degradation upon prolonged exposure to elevated temperatures, potentially altering the material’s adhesive properties [36,37]. However, in the present study, the total-etch procedure was applied for all tested cements in order to minimize the number of experimental variables and to ensure comparability between groups. Further investigations are needed to evaluate the thermal stability of acidic monomers in resin cements and to determine how temperature-related degradation may influence their adhesive potential and long-term bonding performance.

The influence of the resin matrix composition on the thermal behavior of luting cements appears to be complex and material-dependent. Although the literature emphasizes that monomer structure, viscosity and polymer network characteristics may affect resistance to temperature-related changes, the results from the present study indicate that such relationships cannot be directly generalized. The tested cements differed in their responses to elevated storage temperature, and no single compositional factor could reliably account for all observed variations in bond strength. This suggests that the thermal stability of dual-cure cements is determined by a combination of components, including monomers, initiators, and other formulation-dependent factors, rather than by the presence of any individual compound alone. Further material-specific investigations are required to clarify the contribution of particular constituents to temperature-related changes in bonding performance.

In summary, the results of this study, together with evidence from previous reports, demonstrate that the storage temperature of resin cements can significantly affect their bonding performance. It seems that the extent of these changes depends on the material’s chemical composition and, in particular, on the stability of its initiator system. Benzoyl peroxide (BPO), a key component of chemically cured cements, appears to be the most thermally sensitive compound, and its accelerated decomposition at elevated temperatures may lead to premature polymerization and a consequent reduction in bond strength. Materials employing alternative initiator systems, such as MaxCem Elite, exhibited greater thermal resilience, and, in some cases, improved adhesive performance at higher temperatures.

These findings underscore the importance of proper storage and transport of composite cements, particularly in warm climates where exposure to elevated temperatures is more likely. The balance between chemical and photo-initiated polymerization should be considered, since the chemical-curing component remains essential in areas with limited light access. Finally, future studies should also focus on the stability of acidic monomers incorporated into self-adhesive and universal resin cements, as well as on their long-term adhesive potential under varying environmental conditions.

## 5. Conclusions

1. The storage temperature of dual composite cements significantly affects their bond strength to dentin. Cements with benzoyl peroxide as an initiator, when stored at 50 °C, exhibited lower shear bond strength (SBS) than those stored at 25 °C, confirming their sensitivity to adverse thermal conditions.
2. Despite the lack of statistical significance, light-activated cements (LC) consistently demonstrated numerically higher shear bond strength (SBS) than chemically cured cements (CC) across all storage temperatures. This pattern suggests that light activation may play a key role in ensuring optimal bond strength of the material to dentin.
3. MaxCem Elite exhibited the highest temperature stability due to its proprietary redox initiator system.
4. The analysis of the results indicates that both the type of activation and the chemical composition of the cement determine its behavior under elevated temperature conditions.
5. In clinical practice, it is recommended to store cements under conditions in accordance with the manufacturer’s instructions and to use light activation wherever possible to increase the durability of the bond with tooth tissues.

## Figures and Tables

**Table 1:** The composition of the investigated cements.

Cement	Polymer Matrix/Monomers	Fillers/Initiators	Filler Content
Multilink Automix (Ivoclare Vivadent)	Dimethacrylates (22–26%) HEMA (6–7%) Benzoyl peroxide (<1%) Stabilizers Pigments	Ytterbium trifluoride	Not specified in SDS
EnaCem HF (Micerdent)	Tetramethylene dimethacrylate (2.5–10%) Dibenzoyl peroxide (<2.5%) Diphenyl (2,4,6-trimethylbenzoyl)phosphine oxide (<2.5%)	Not specified in SDS	Not specified in SDS
MaxCem Elite (Kerr)	1,6-hexanediyl bismethacrylate (5–10%) 2-hydroxy-1,3-propanediyl bismethacrylate (5–10%) 7,7,9 (or 7,9,9)-trimethyl-4,13-dioxo-3,14-dioxa-5,12-diazahexadecane-1,16-diyl bismethacrylate (1–5%) 3-trimethoxysilylpropyl methacrylate (1–5%)	Barium aluminoborosilicate glass (30–60%) Ytterbium fluoride (10–30%) Fumed silica (1–5%)	Not specified in SDS
Bifix Hybrid Abutment (VOCO)	Urethanedimethacrylate (5–10%) Glycerindimethacrylate (5–10%) BIS-GMA (5–10%) Acidic adhesive monomer (5–10%) Hydroxypropyl methacrylate (2.5–5%) Benzoyl peroxide (≤2.5%) Catalyst, unspecified (1–2.5%), Initiator, unspecified	Not specified in SDS	Not specified in SDS

**Table 2:** Materials used in the study and application procedures.

Material	Type/Characteristics	Manufacturer (Country)	Application Procedure
37% phosphoric acid (Blue Etch)	Etching gel for enamel and dentin	Arkona Dental, Nasutów, Poland	Applied to the dentin surface for 15 s, rinsed thoroughly with water, and gently air-dried, leaving a slightly moist surface
Solo Bond Plus	Single-component, light-curing etch-and rinse adhesive system	VOCO GmbH, Cuxhaven, Germany	A uniform layer was applied and rubbed for approximately 20 s, gently air-thinned, and light-cured for 10 s using an LED unit (≥1000 mW/cm^2^)
Bifix Hybrid Abutment	Dual-cure composite cement	VOCO GmbH, Cuxhaven, Germany	The cement was applied onto the dentin surface covered with an adhesive layer; the element was seated, excess cement removed, left for 90 s, and then light-cured for 20 s (LC) or allowed to self-polymerize (CC)
MaxCem Elite	Self-adhesive dual-cure composite cement	Kerr, Orange, CA, USA	The cement was applied without prior adhesive use; the element was positioned, excess cement removed, left for 90 s, and then light-cured for 20 s (LC) or allowed to self-polymerize (CC)
EnaCem HF	Dual-cure composite cement	Micerium, Avegno, Italy	The cement was applied onto the dentin surface pretreated with an adhesive, left for 90 s, and then light-cured for 20 s (LC) or allowed to self-polymerize (CC)
Multilink Automix	Dual-cure composite cement	Ivoclar Vivadent, Schaan, Liechtenstein	The cement was applied onto the dentin surface covered with an adhesive layer, excess cement removed, left for 90 s, and light-cured for 20 s (LC) or allowed to self-polymerize (CC)

## Data Availability

The original contributions presented in this study are included in the article/Supplementary Materials. Further inquiries can be directed to the corresponding author.

## Supplementary Materials

The following supporting information can be downloaded at https://www.mdpi.com/article/10.3390/ma19040718/s1. Table S1: Descriptive statistics of shear bond strength (SBS, MPa); Table S2: Failure mode.
