# Supplementary material for: Effect of Pre-Conditioning Temperature and Method of Curing on the Shear Bond Strength of Dual-Cure Composite Cements to Dentin
Source: Materials (Basel). 2026 Feb 13;19(4):718. doi: 10.3390/ma19040718 (PMC12942536; doi:10.3390/ma19040718)
Supplement: Supplementary file 1 [file materials-19-00718-s001.zip › materials-4105913-supplementary.pdf]

materials

MDPI

Supporting Information

Effect of Pre-Conditioning Temperature and Method of Curing on the Shear Bond Strength of Dual-Cure Composite Cements to Dentin

Joanna Giełzak [1], Agata Szczesio-Wlodarczyk [2,*] and Kinga Bociong [3,*]

[1] Department of Prosthodontics, Medical University of Lodz, 92-213 Łódź, Poland; joanna.gielzak@umed.lodz.pl
[2] Laboratory of Materials Research, Medical University of Lodz, 92-213 Łódź, Poland
[3] Department of General Dentistry, Medical University of Lodz, 92-213 Łódź, Poland
* Correspondence: agata.szczesio@umed.lodz.pl (A.S.-W.); kinga.bociong@umed.lodz.pl (K.B.)

Table S1. Descriptive statistics of shear bond strength (SBS, MPa).

Cement	Temperature	Curing Mode	Min	Max	Mean	SD	Median	IQR
Bifix	25 °C	CC	7.35	10.30	8.89	0.94	8.74	1.03
	25 °C	LC	8.02	14.6	10.61	2.04	10.5	1.68
	50 °C	CC	3.02	6.11	4.39	0.99	4.58	1.38
	50 °C	LC	7.42	12.2	9.97	1.79	10	2.56
EnaCem	25 °C	CC	8.02	10.40	9.23	0.94	9.23	0.94
	25 °C	LC	9.36	14.50	11.57	2.15	11.1	2.15
	50 °C	CC	3.42	5.73	4.54	0.64	4.53	0.64
	50 °C	LC	5.01	12.00	8.45	2.37	7.98	2.37
MaxCem	25 °C	CC	7.55	12.20	9.39	1.38	9.44	1.44
	25 °C	LC	8.89	12.70	10.37	1.16	10.3	1.47
	50 °C	CC	9.13	13.00	10.86	1.37	10.8	2.13
	50 °C	LC	8.32	12.50	10.81	1.50	10.4	2.60
Multilink	25 °C	CC	6.8	10.90	8.93	1.52	9.01	2.77
	25 °C	LC	8.85	13.30	10.36	1.31	9.88	1.24
	50 °C	CC	4.57	7.26	5.69	1.12	5.38	2.03
	50 °C	LC	5.61	8.64	7.30	1.17	7.63	2.23

Table S2. Failure mode.

Cement	Temperature	Curing Mode	Adhesive Failure [%]	Mixed Failure [%]
Bifix	25 °C	CC	100	0
	25 °C	LC	100	0
	50 °C	CC	100	0
	50 °C	LC	100	0
EnaCem	25 °C	CC	100	0
	25 °C	LC	90	10
	50 °C	CC	100	0
	50 °C	LC	100	0
MaxCem	25 °C	CC	100	0
	25 °C	LC	100	0
	50 °C	CC	100	0
	50 °C	LC	100	0
Multilink	25 °C	CC	100	0
	25 °C	LC	100	0
	50 °C	CC	100	0
	50 °C	LC	100	0

 https://doi.org/10.3390/ma19040718
